# Interactions among type I and type II interferon, tumor necrosis factor, and β-estradiol in the regulation of immune response-related gene expressions in systemic lupus erythematosus

**DOI:** 10.1186/ar2584

**Published:** 2009-01-03

**Authors:** Hooi-Ming Lee, Toru Mima, Hidehiko Sugino, Chieko Aoki, Yasuo Adachi, Naoko Yoshio-Hoshino, Kenichi Matsubara, Norihiro Nishimoto

**Affiliations:** 1 Laboratory of Immune Regulation, Graduate School of Frontier Biosciences, Osaka University, 1-3 Yamada-Oka, Suita City, Osaka 565-0871, Japan; 2 DNA Chip Research Incorporated, 1-1-43 Suehirocho, Tsurumi-ku, Yokohama, Kanagawa 230-0045, Japan

## Abstract

**Introduction:**

Systemic lupus erythematosus (SLE) is a prototypical autoimmune disease characterized by various clinical manifestations. Several cytokines interact and play pathological roles in SLE, although the etiopathology is still obscure. In the present study we investigated the network of immune response-related molecules expressed in the peripheral blood of SLE patients, and the effects of cytokine interactions on the regulation of these molecules.

**Methods:**

Gene expression profiles of peripheral blood from SLE patients and from healthy women were analyzed using DNA microarray analysis. Differentially expressed genes classified into the immune response category were selected and analyzed using bioinformatics tools. Since interactions among TNF, IFNγ, β-estradiol (E2), and IFNα may regulate the expression of interferon-inducible (IFI) genes, stimulating and co-stimulating experiments were carried out on peripheral blood mononuclear cells followed by analysis using quantitative RT-PCR.

**Results:**

Thirty-eight downregulated genes and 68 upregulated genes were identified in the functional category of immune response. Overexpressed IFI genes were confirmed in SLE patient peripheral bloods. Using network-based analysis on these genes, several networks including cytokines – such as TNF and IFNγ – and E2 were constructed. TNF-regulated genes were dominant in these networks, but *in vitro *TNF stimulation on peripheral blood mononuclear cells showed no differences in the above gene expressions between SLE and healthy individuals. Co-stimulating with IFNα and one of TNF, IFNγ, or E2 revealed that TNF has repressive effects while IFNγ essentially has synergistic effects on IFI gene expressions *in vitro*. E2 showed variable effects on IFI gene expressions among three individuals.

**Conclusions:**

TNF may repress the abnormal regulation by IFNα in SLE while IFNγ may have a synergistic effect. Interactions between IFNα and one of TNF, IFNγ, or E2 appear to be involved in the pathogenesis of SLE.

## Introduction

Systemic lupus erythematosus (SLE) is a prototypical autoimmune disease characterized by multiple organ damage, high titers of autoantibodies, and various clinical manifestations [1]. Numerous disorders in the immune system and abnormalities in cytokine productions have been described in patients with SLE. The exact pathological mechanisms are still obscure, however, and the roles of the cytokines are not well understood. High levels of TNF, type I interferon, and type II interferon in the sera of patients with SLE have been reported [2-4]. On the other hand, an impaired production of IL-12 by T lymphocytes from SLE patients *in vitro *has also been observed [5,6]. Cytokines are pleiotropic in their biological activity, and it is known that our immunity is regulated by highly sophisticated cytokine networks. Comprehending the pathological roles of these abnormally induced cytokines and immunoregulatory networks of cytokines in SLE patients is therefore important so that appropriate treatment can be offered.

The microarray is a powerful tool to exhaustively investigate the gene expressions of autoimmune diseases that have complex pathogenesis and heterogeneous manifestations, such as SLE. So too are the various databases and bioinformatics tools such as gene ontology analysis, which can functionally categorize genes, or network-based analysis to investigate molecule interactions [7]. These tools have proven useful to further analyze the enormous data from microarray analysis, providing several new findings [8].

Most microarray analyses in SLE have been performed using peripheral blood mononuclear cells (PBMCs) while recent studies provide strong evidence that IFN-related genes are overexpressed in SLE patients [9-13]. In the present study, to investigate the abnormal immune system in SLE, we focused on genes in the functional category of immune response differentially expressed in SLE patients compared with healthy individuals. Our results using SLE whole blood showed definite overexpression of IFN-regulated genes in this category. As molecules in the immune response category are always communicating with each other, we performed a network-based analysis to identify aberrant regulations or interactions among differentially expressed molecules observed in this study. We also investigated the effect of interactions between IFNα and one of TNF, IFNγ, or β-estradiol (E2) on the expression of these molecules.

## Materials and methods

### Patients and healthy individuals

Eleven patients (all women, median age 35 years, range 27 to 72 years) with SLE fulfilled by the diagnostic criteria of the American College of Rheumatology [14] and six healthy women were enrolled in the present study after obtaining their written informed consent. The study was approved by the Ethical Committee of Osaka University Medical School for clinical studies on human subjects.

The majority of the SLE patients (n = 10) were treated with <20 mg/day prednisolone. Three of these 10 patients were treated with one of cyclosporine, azathioprine, or methotrexate in combination with prednisolone, respectively. The remaining patient was treated with >20 mg/day prednisolone.

The median disease activity score of SLE patients based on the SLE Disease Activity Index 2000 instrument [15] was 10 (range 6 to 24). Two patients had very active states (SLE Disease Activity Index 2000 score >12) while the other patients had active states (SLE Disease Activity Index 2000 score = 4 to 12). The median of the assessment based on the BILAG index [16] was 4 (range 1 to 13).

Meanwhile, the median of the total white blood cells for the patients was 6,160 (range 4,840 to 12,230). The median of the total number (proportion) of neutrophils was 4,919 (80.0%) (range 3,640 to 9,674, 75.2% to 90.1%), and that of lymphocytes was 838 (11.8%) (range 480 to 1,517, 6.6% to 20.5%).

### GeneChip microarray and data analysis

Peripheral blood was collected directly into PAXGene tubes (Qiagen, Valencia, CA, USA). Total RNA was extracted using the PAXGene Blood RNA kit with the optimal on-column DNase digestion (Qiagen). Amino allyl RNA (aRNA) was synthesized from 1 μg total RNA using the Amino Allyl MessageAmp™ aRNA kit (Ambion, Austin, TX, USA). Five micrograms of aRNA from each sample (11 SLE patients and six healthy control individuals) and the equivalent quantity of reference aRNA from a mixture of RNA extracted from peripheral blood of 12 healthy women were subjected to Cy3 and Cy5 labeling, respectively. Both labeled aRNAs were mixed in equal amounts and were hybridized with the oligonucleotide-based DNA microarray AceGene (HumanOligoChip30K; DNA Chip Research, Yokohama, Japan), which contained about 30,000 human genes.

The microarrays were scanned using ScanArray Lite (PerkinElmer, Boston, MA, USA) and the signal values were calculated using the DNASIS Array (Hitachi Software Engineering, Tokyo, Japan) according to the manufacturer's instructions. The intensities of no-probe spots were used as the background. The median and standard deviation of background levels were calculated. Genes whose intensities were less than the median plus two standard deviations of the background level were identified as null. The Cy3/Cy5 ratios of all spots on the DNA microarray were normalized by the global ratio median. Genes with at least 80% good data across each group of samples were selected for further analysis. The microarray data have been deposited in NCBIs Gene Expression Omnibus [GEO:GSE12374].

### Gene ontology and network-based analysis

Genes identified to be differentially expressed by >10% according to the microarray analysis with a median signal intensity difference of at least 100 between the SLE patient and healthy individual groups (in order to reduce errors pertaining to low-level expression at close to noise level) were functionally categorized using Expression Analysis Systematic Explorer version 2.0 bioinformatics software [17,18]. Interactions among the differentially expressed genes in the functional category of immune response were investigated through the use of Ingenuity Pathway Analysis version 5.5 [19]. Networks generated by less than five uploaded genes were excluded from the analysis.

### Stimulation of peripheral blood mononuclear cells

To assess TNF signaling, PBMCs from six patients diagnosed with SLE and from three healthy individuals were utilized. All PBMCs used in the experiments were isolated from heparinized whole blood using a Ficoll-Paque™ Plus (GE Healthcare Biosciences, Uppsala, Sweden) gradient centrifugation according to the manufacturer's recommendations. The cells were incubated in RPMI 1640 with 10% heat-inactivated fetal bovine serum and TNF (20 ng/ml) in a carbon dioxide incubator at 37°C for 24 hours.

To examine the effects of interactions between IFNα and one of TNF, IFNγ, or E2 on interferon-inducible (IFI) genes, we performed co-stimulating experiments on PBMCs. The PBMCs isolated from three healthy women were cultured with 20 ng/ml TNF, 15 ng/ml IFNγ, 2 ng/ml E2, and 500 U/ml IFNα or null, and were co-stimulated with TNF and IFNα, with IFNγ and IFNα, or with E2 and IFNα. PBMCs were cultured at a final concentration of 1.5 × 10^6 ^cells/ml.

TNF [GenBank:CAA26669] and IFNγ [GenBank:AAB59534] were purchased from R&D Systems (Minneapolis, MN, USA). IFNα [GenBank:NP_000596] and E2 were purchased from PBL Biomedical Laboratories (Piscataway, NJ, USA) and Sigma (St Louis, MO, USA), respectively.

### Preparation of cDNA and quantitative RT-PCR

Total RNA from the PBMCs was extracted using the RNeasy Mini Kit (Qiagen) according to the manufacturer's instructions. One microgram of RNA was reverse-transcribed into cDNA using 2.5 μM random hexamers and 125 units MuLV reverse transcriptase (Applied Biosystems, Foster City, CA, USA) in a 100 μl reaction mixture. Four microliters of the twofold-diluted cDNA products were amplified in a 25 μl reaction mixture containing TaqMan Universal Master Mix and each TaqMan probes (Applied Biosystems). The assay identification numbers for the probes are presented in Table 1.

**Table 1:** Assay identification numbers for probes

Probe	Identification number
CD40	Hs01002913_m1
CD1C	Hs00233509_m1
CD14	Hs00169122_g1
Chemokine (C-C motif) receptor 7 (CCR7)	Hs00171054_m1
IL12B	Hs00233688_m1
IL-4 receptor (IL4R)	Hs00166237_m1
Prostaglandin E synthase (PTGES)	Hs00610420_m1
Interferon-induced protein with tetratricopeptide repeats 1 (IFIT1)	Hs01911452_m1
Interferon-induced protein with tetratricopeptide repeats 3 (IFIT3)	Hs00382744_m1
Interferon-induced protein with tetratricopeptide repeats 5 (IFIT5)	Hs00202721_m1
Interferon, alpha-inducible protein 6 (IFI6)	Hs00242571_m1
Interferon, gamma-inducible protein 16 (IFI16)	Hs00194261_m1
Interferon, alpha-inducible protein 27 (IFI27)	Hs00271467_m1
Interferon, gamma-inducible protein 30 (IFI30)	Hs00173838_m1
Interferon-induced protein 35 (IFI35)	Hs00413458_m1
Interferon induced transmembrane protein 1 (IFITM1)	Hs01652522_g1
Interferon-stimulated gene, 15 kDa (ISG15)	Hs00192713_m1
Interferon regulatory factor 7 (IRF7)	Hs00242190_g1
2',5'-oligoadenylate synthetase 1 (OAS1)	Hs00242943_m1
2',5'-oligoadenylate synthetase-like (OASL)	Hs00388714_m1
Guanylate binding protein 1 (GBP1)	Hs00266717_m1
Guanylate binding protein 2 (GBP2)	Hs00269759_m1
IL8RA	Hs00174146_m1
C-type lectin domain family 4, member E (CLEC4E)	Hs00372017_m1
TNFα-induced protein 6 (TNFAIP6)	Hs00200180_m1

The real-time PCR was performed in a 96-well optical plate with the Applied Biosystems 7500 real-time PCR system under the following cycling conditions: 2 minutes at 50°C (one cycle), 10 minutes at 95°C (one cycle), 15 seconds at 95°C and 1 minute at 60°C (40 cycles). For each gene (performed in duplicate for each sample), cycle threshold (Ct) values were determined from the linear region of the amplification plot and were normalized by subtracting the Ct value of GAPDH (generating a ΔCt value). The response to the cytokines or E2 was determined by subtracting the ΔCt value for the time-matched control from the ΔCt value for the stimulated sample (ΔΔCt value). The fold change was subsequently calculated using the formula 2ΔΔCt (where ΔΔCt was converted to an absolute value).

### Statistical analysis

The unpaired Mann-Whitney test was used to determine statistically significant differences in the mRNA expression levels between the SLE patient and healthy individual groups. The criterion for the statistical significance was *P *< 0.05.

## Results

### Immune response-related genes identified by gene ontology analysis

Thirty-eight downregulated genes and 68 upregulated genes were categorized into the functional category of immune response. Most of the 68 upregulated genes were interferon regulated – including 17 IFI genes such as interferon-induced protein with tetratricopeptide repeats (IFIT) 1, 2',5'-oligoadenylate synthetase 1 (OAS1), 2',5'-oligoadenylate synthetase-like (OASL), interferon-stimulated gene, 15 kDa (ISG15), and interferon regulatory factor 7 (IRF7) that have been reported as overexpressed in the PBMCs of SLE.

### Network-based analysis on the downregulated or upregulated genes in the functional category of immune response

**Figure 1:**
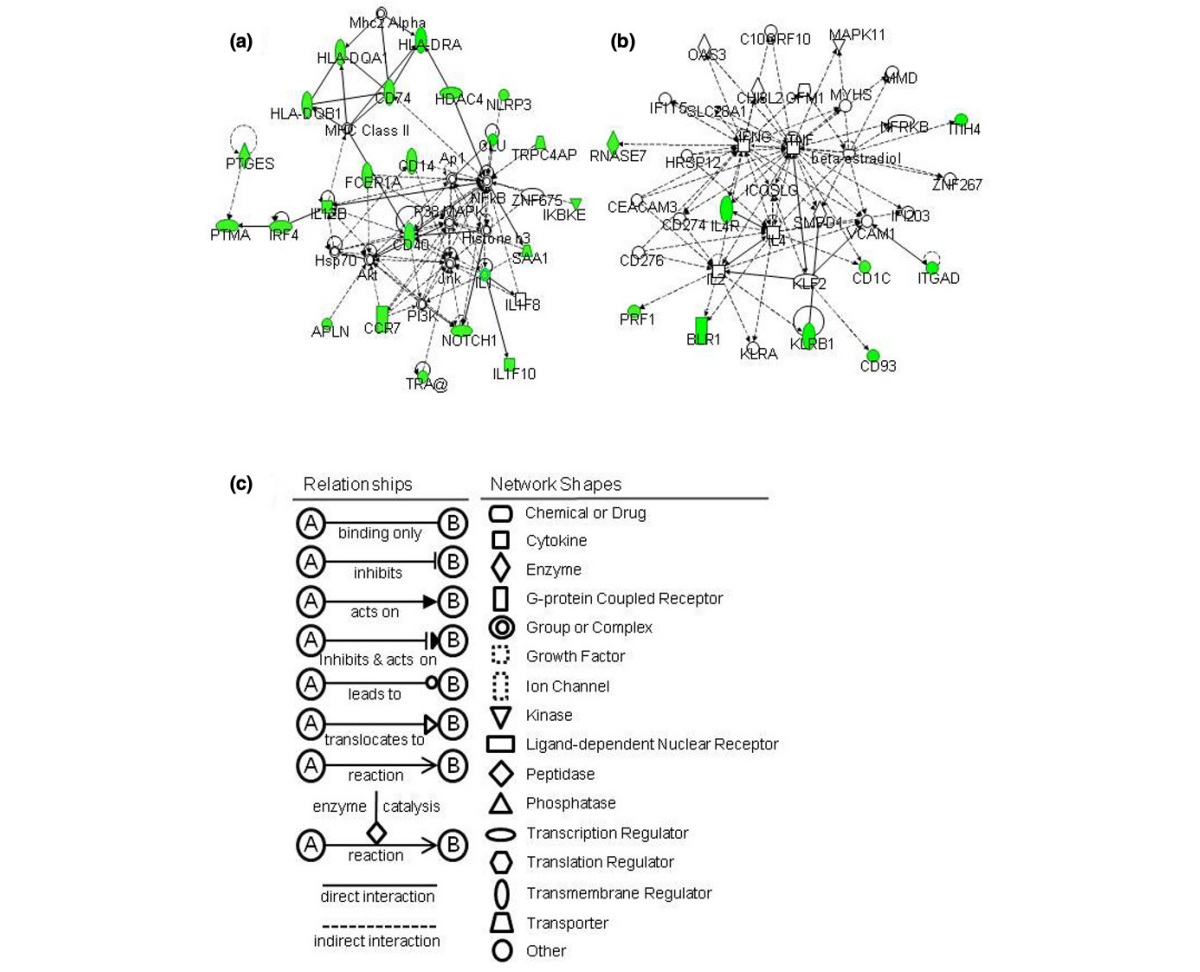
**Network-based analysis of downregulated genes in the functional category of immune response**. **(a) **Network 1 and **(b) **Network 2 constructed by downregulated genes. **(c) **Network graphical representation. Genes or gene products are represented as individual nodes whose shapes represent the functional class of gene products. The biological relationship between the two nodes is represented as an edge (line). All edges are supported by at least one reference from the literature stored in the Ingenuity Pathways Knowledge Base (IPKB). Genes in colored nodes were found over-represented in the functional category of immune response. Genes in uncolored nodes were not found over-represented but were depicted by the computationally generated networks on the basis of evidence stored in the IPKB indicating a strong biologic relevance to that network.

There were two networks represented by the downregulated genes. Twenty-three out of the 38 downregulated genes were included in the first network, including p38 mitogen-activated protein kinase (MAPK) complex and NFκB complex depicted at the center of Figure 1a. p38 MAPK is phosphorylated in response to inflammatory cytokines including IL-1β [20] and TNF. Phosphorylated p38 MAPK contributes to the activation of NFκB, which regulates the gene expression of various cytokines, chemokines and adhesion molecules [21]. Although TNF was not identified in this network, we found that most of the molecules were TNF-regulated – including cell surface antigens (CD40, CD14, CD1C), chemokine (C-C motif) receptor 7, and acute phase proteins such as serum amyloid A_1 _and apelin. These data, together with a previous report of increased TNF levels in the serum of SLE patients [2], suggested that an abnormality in TNF signaling might exist. Meanwhile, a cluster of MHC class II genes consisting of HLA-DRA, HLA-DQA1, HLA-DQB1, and CD74 (also known as HLA-DRG) were also identified in this network. The second network, composed of nine downregulated genes, implied that there were interactions among TNF, IFNγ, IL-2, IL-4, and E2 (Figure 1b).

**Figure 2:**
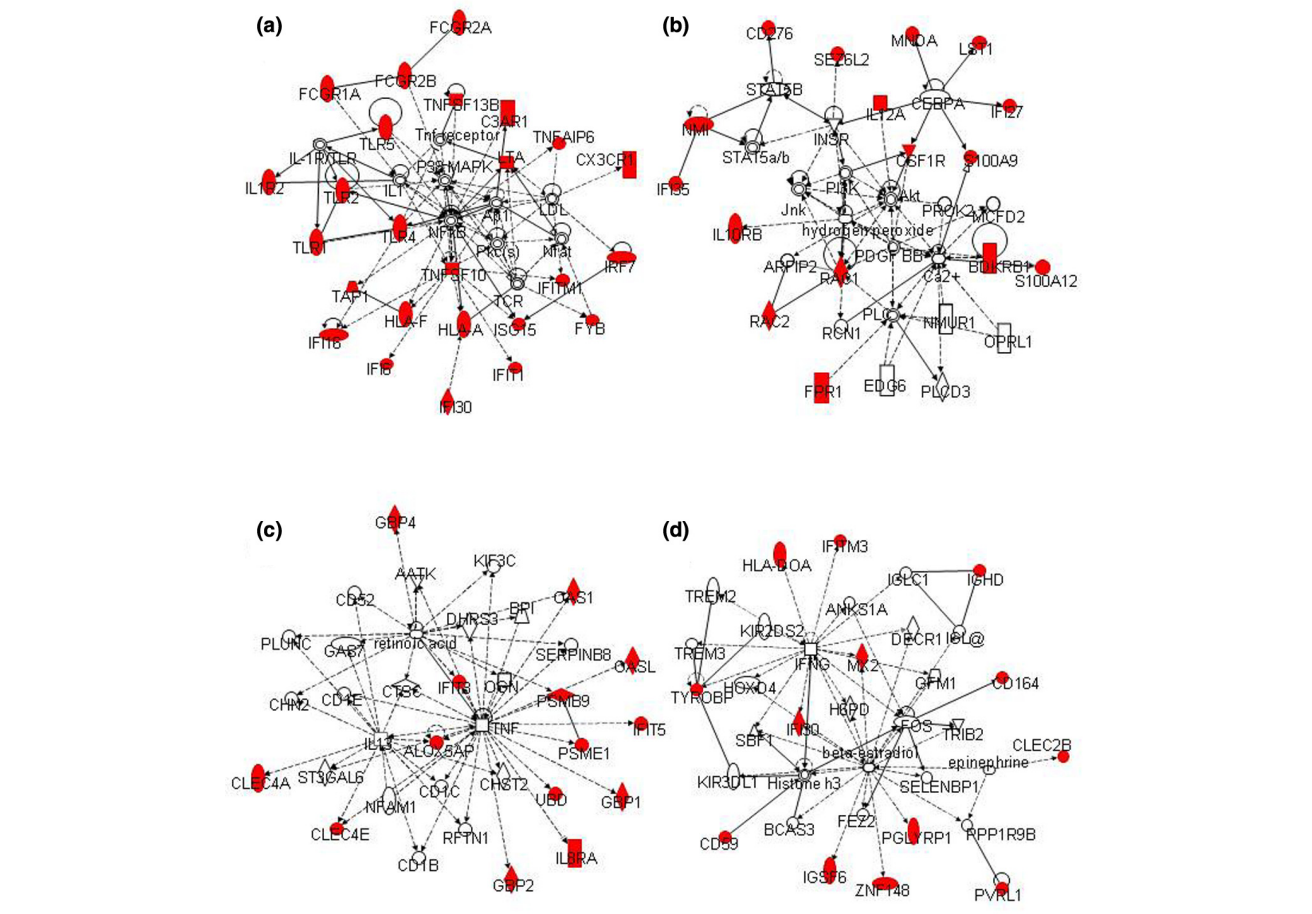
**Network-based analysis of upregulated genes in the functional category of immune response**. **(a) **Network 1, **(b) **Network 2, **(c) **Network 3, and **(d) **Network 4 constructed by upregulated genes.

Our analysis found only four networks represented by the upregulated molecules. The first network, constructed by 25 upregulated molecules, was the network with p38 MAPK, NFκB, and TNF receptor depicted at the center of Figure 2a. A cluster of the Toll-like receptor (TLR) family (that is, TLR1, TLR2, TLR4, and TLR5) and another cluster of Fcγ receptors (FcγRs) were identified in this network. The two clusters were indirectly connected through p38 MAPK and NFκB, suggesting there may be functional interactions among these molecules through this pathway. This network was overlapped with the fourth network, whose central molecules were IFNγ and E2 (Figure 2d). There were nine IFI molecules found in the first and fourth networks. The second network was represented by Akt and a calcium ion at the center (Figure 2b), while the third network was mainly attributed to TNF (Figure 2c). We found that two IFI molecules were included in the second network, and that seven out of the 14 upregulated molecules that constructed the third network were IFI molecules.

Gathering the above results, TNF, IFNγ, and E2 were depicted by both downregulated and upregulated molecules in the networks. As most of the genes in the immune response were TNF regulated, we performed stimulating experiments on the PBMCs of SLE patients and healthy individuals to assess the TNF regulation on the immune response-related molecules in SLE. On the other hand, although the expression of IFNα was not upregulated and was not depicted in networks related to TNF, IFNγ, or E2, IFI molecules were found ranging over the four networks. Furthermore, it has been reported that there exist elevated levels of type I interferon in the SLE serum. Type I interferon therefore appears to have complicated interactions with various cytokines and E2. This encouraged us to further examine the effects of interactions between IFNα and one of TNF, IFNγ, or E2 on IFI gene expression.

### Gene expression profiles of peripheral blood mononuclear cells by TNF stimulation for SLE patients and healthy individuals

**Figure 3:**
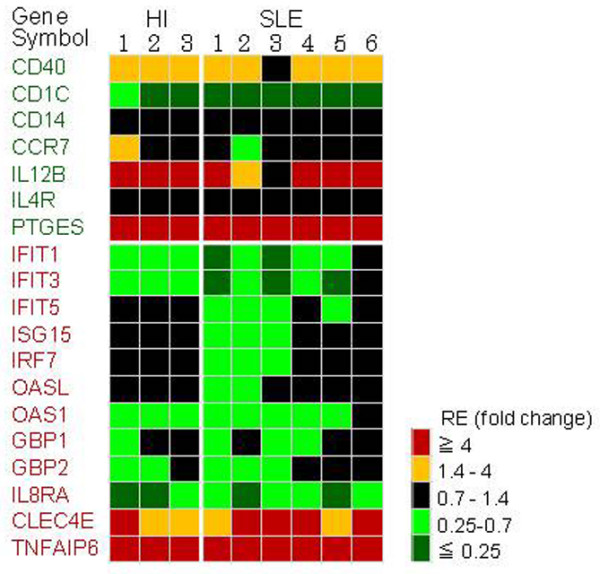
**Effect of TNF stimulation on gene expression in healthy individuals and systemic lupus erythematosus patients**. Peripheral blood mononuclear cells (PBMCs) from six systemic lupus erythematosus (SLE) patients and three healthy individuals (HI) were isolated and stimulated for 24 hours in the absence and presence of 20 ng/ml TNF. The relative mRNA expressions (RE) compared between TNF-stimulated and nonstimulated control individuals were measured using quantitative RT-PCR. The RE of seven downregulated genes (highlighted in green) and 12 upregulated genes (highlighted in red) are designated by five colors as shown. See Table 1 for gene identification.

Seven downregulated genes (CD40, CD1C, CD14, chemokine (C-C motif) receptor 7, IL12B, IL-4 receptor, and prostaglandin E synthase) and 12 upregulated genes (IFIT1, IFIT3, IFIT5, ISG15, IRF7, OASL, OAS1, guanylate binding protein (GBP) 1, GBP2, IL8RA, C-type lectin domain family 4 member E, and TNFα-induced protein 6), all of which were TNF regulated, were selected and their mRNA expressions upon TNF stimulation were measured by quantitative RT-PCR. All of the genes selected showed essentially the same responses to TNF stimulation on PBMCs independent of the individual (Figure 3). CD40, IL12B, prostaglandin E synthase, C-type lectin domain family 4 member E, and TNFα-induced protein 6 were upregulated, while CD1C, IFIT1, IFIT3, OAS1, and IL8RA were downregulated upon TNF stimulation in both SLE patients and healthy individuals.

The *in vivo *gene expression profiles of SLE, however, were different from the results of *in vitro *PBMC stimulation by TNF. For example, CD40 was downregulated *in vivo *but was upregulated upon TNF stimulation *in vitro*. Meanwhile, IFI genes such as IFIT1, IFIT3, OAS1, ISG15 and IRF7, and IL8RA were upregulated *in vivo*, but IFIT1, IFIT3, OAS1 and IL8RA were downregulated, while ISG15 and IRF7 showed almost no response to TNF *in vitro*. These data suggest that other soluble factors might be involved in the regulation on the gene expression. Indeed, high levels of interferon in SLE serum have been suggested to cause overexpression of IFI genes [22]. Interestingly, we not only found that TNF had repressive effects on IFI genes IFIT1, IFIT3, IFIT5, ISG15, and IRF7 expression, but that the effect was significantly stronger on SLE patients' PBMCs than those of healthy individuals. This result may be caused by the differences in the baseline expressions where IFI genes were overexpressed *in vivo *in SLE patients.

### Repressive effect of TNF on interferon-inducible gene expressions in peripheral blood mononuclear cells *in vitro*

The expression of 15 IFI genes (IFIT1, IFIT3, IFIT5, IFI6, IFI16, IFI27, IFI30, IFI35, interferon-induced transmembrane protein 1, ISG15, IRF7, OAS1, OASL, GBP1, and GBP2) in PBMCs upon stimulation were measured. All of these genes were upregulated upon IFNα stimulation, while only some were upregulated by IFNγ (data not shown). On the other hand, TNF also showed a repressive effect on the expressions of most IFI genes in PBMCs *in vitro *in this experiment.

**Figure 4:**
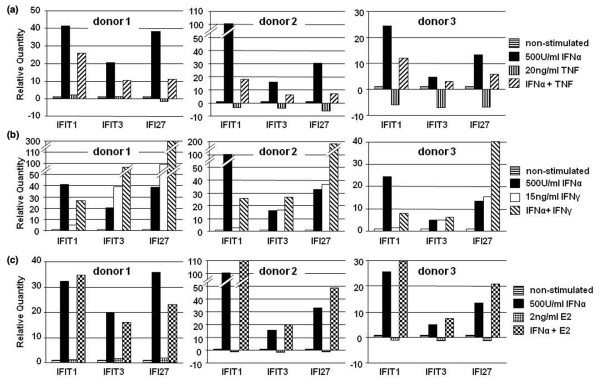
**Effect of cytokines or β-estradiol on the expressions of interferon-inducible genes**. Peripheral blood mononuclear cells from three healthy donors were cultured with the indicated cytokines for 24 hours. RNA was analyzed by quantitative RT-PCR as described in Materials and methods. Relative expression of the indicated genes – interferon-induced protein with tetratricopeptide repeats 1 (IFIT1), interferon-induced protein with tetratricopeptide repeats 3 (IFIT3), and interferon alpha-inducible protein 27 (IFI27) – compared with their nonstimulated cultures is shown. Each bar represents the mean value of duplicate wells as compared with the nonstimulated control. Downregulated genes were arbitrarily assigned a negative value.

The relative expressions of three of the representative genes (that is, IFIT1, IFIT3, and IFI27) from three women are shown in Figure 4. A remarkable suppression was observed through the TNF and IFNα co-stimulating experiment (Figure 4a). On the other hand, there was synergism between IFNγ and IFNα on IFI gene expressions, although with some exceptions like IFIT1 (Figure 4b). IFIT1 was downregulated upon IFNγ and IFNα co-stimulation, unlike stimulation with IFNα alone. E2 showed no significant or consistent interaction with IFNα for most of the IFI genes. Inconsistent responses to E2 stimulation, however, were observed among the three healthy donors on IFI27. E2 tended to downregulate IFI27 expression in one donor but upregulated expression in the other two donors (Figure 4c).

To test a hypothesis that TNF decreases IFI gene expression through suppressing IFNα production, we examined the effect of TNF or IFNα on IFNα mRNA expression. Its expression was too low to be measured and there were no significant changes in TNF, IFNα, or TNF + IFNα 24-hour-stimulated PBMCs.

## Discussion

To identify the molecules involved in the aberrant immune system of SLE, we compared the gene expression profiles of peripheral blood between SLE patients and healthy individuals using microarray technology followed by gene ontology analysis. Most previously reported SLE studies utilizing microarray analysis have used PBMCs, but in the present study we used whole blood from SLE patients to exhaustively analyze the gene expression profiles of immune response-related molecules *in vivo*. Despite an additional proportion of granulocytes (mainly neutrophils), our results showed that there was an overexpression of several interferon-regulated genes. This result was in agreement with a previous report showing that peripheral blood from SLE patients had remarkably homogeneous gene expression patterns with an overexpression of IFI genes [10], and confirms the involvement of interferon in SLE.

Since the immune system is regulated by an elaborate network, interactions among the downregulated genes and the upregulated genes of the immune response category were further investigated by utilizing network-based analysis. A cluster of the TLR family (that is, TLR1, TLR2, TLR4, and TLR5) and another cluster of FcγRs were upregulated and depicted in the same network, which had p38 MAPK and NFκB at the center. Our finding that FcγR genes were overexpressed in the peripheral blood of SLE patients is novel, although the overexpression of TLR genes has been recently reported [23]. Furthermore, this is the first report showing that these clusters possibly interact with each other through p38 MAPK and NFκB signaling pathways in a network, and consequently contribute to SLE. Indeed, it has been shown that FcγRIIb is a gene susceptible to SLE both in humans and mice [24]. Means and Luster have reported that a functional interaction between TLR9 and CD32 (also known as FcγRIIa) may be involved in the pathogenesis of SLE, and they also have suggested the possibility that TLR7 may activate cells through similar pathways [25]. Although in our study overexpression of DNA-recognizing TLR9, which has been suggested to be triggered by immune complexes containing DNA in SLE [26,27], was not statistically significant according to the rank test, seven out of the 11 SLE patients showed upregulated expressions of TLR9. In addition, TLR1, TLR2, TLR4, and TLR5 – which serve to recognize bacterial components such as lipopolysaccharide or lipopeptides [28,29] – were also upregulated. Our network-based analysis therefore suggested the hypothesis that the interaction between TLRs and FcγRs is involved in the pathogenesis of SLE.

We additionally found that networks whose central molecule was TNF, IFNγ, or E2 were represented by both the downregulated genes and the upregulated genes in the functional category of immune response. This observation suggested that TNF, IFNγ, or E2 may be involved in the abnormal expressions of both downregulated and upregulated genes in the immune response. Indeed, the elevated level of some cytokines such as TNF and interferon in the sera of SLE patients has been reported [2,4,30,31]. Although our data did not show a significant increase in the gene expressions of TNF, IFNγ, or IFNα in themselves according to rank test, more than one-half of the SLE patients' individual data showed an increase in the TNF gene expression in our study (data not shown). For IFNα, the expression was not increased in the peripheral blood but it may be produced at the other site. Siegal and colleagues have demonstrated that purified interferon-producing cells were CD4^+^CD11c^- ^type 2 dendritic cell precursors, which produce 200 to 1,000 times more IFNα than other blood cells after a microbial challenge [32]. E2 is enzymatically synthesized in the ovary, and therefore does not transcript and cannot be detected in peripheral blood in the present study. There is, however, a 10 to 15 times higher frequency of SLE in women during childbearing years, probably due to an estrogen hormonal effect [33]. We therefore believe these results are a good reason to further investigate E2 involvement in SLE pathogenesis.

Concerning the interaction between cytokines, to our knowledge this is the first report showing that TNF has a repressive effect on IFI genes *in vitro*. Although the exact mechanisms of the IFI gene product involvement in SLE pathogenesis are still poorly understood, we suspect that the elevated expression of TNF in SLE reduces the overexpression of IFI genes. Since serum levels of both TNF and IFNα were reportedly elevated in SLE, as mentioned above, it is possible that the increased serum TNF level in SLE is an outcome to compensate the immune system balance altered by IFNα in SLE. Consider that patients with rheumatoid arthritis or Crohn's disease under TNF-blocking therapies can develop autoantibodies to nuclear antigens [34]; therapeutic TNF blockades could thus lead to an exacerbation of certain autoimmune diseases such as SLE and to provoke lupus-like manifestations. Palucka and colleagues reported recently that blocking TNF signaling increases the production of IFNα by plasmacytoid dendritic cells and induces an IFN signature in the blood of arthritis patients [35]. This may be another mechanism for TNF inhibitor to induce the IFN signature. We confirmed that there was no significant effect, however, of TNF on IFNα gene expression in the PBMCs in our experiment. Furthermore, the 500 units/ml IFNα we used for stimulation is obviously a higher amount than endogenously produced IFNα. TNF therefore appeared to directly suppress IFI gene expression in PBMCs. We suggest that the direct suppressive effect of TNF on the IFN signature induced by IFNα, at least, exists in the network regulation of cytokines *in vivo*.

The results of the co-stimulating experiments did not show any strong evidence of a functional interaction between E2 and IFNα on the expression of IFI genes. Inconsistent gene expression patterns were observed in the co-stimulating experiments, possibly due to the hormonal effects of the women donors. The modulation of estrogens on humoral immune response seems to be greatly dependent on its physiological concentration, and E2 is a versatile hormone that plays a wide variety of roles in our body [36]. We therefore cannot exclude the possibility that E2 also plays a significant role in the pathophysiology of SLE.

## Conclusion

TNF may have a counter effect on the abnormal regulation of IFNα on the immune response-related gene expressions, while IFNγ may have a synergistic effect with IFNα in SLE. Interactions between IFNα and one of TNF, IFNγ, or E2 had a suggested involvement in the pathogenesis of SLE.

## Abbreviations

aRNA: amino allyl RNA; Ct: cycle threshold; E2: β-estradiol; FcγR: Fcγ receptor; GBP: guanylate binding protein; HLA: human leukocyte antigen; IFI: interferon-inducible; IFIT: interferon-induced protein with tetratricopeptide repeats; IFN: interferon; IL: interleukin; IRF7: interferon regulatory factor 7; ISG15: interferon-stimulated gene, 15 kDa; MAPK: mitogen-activated protein kinase; MHC: major histocompatibility complex; NFκB: nuclear factor of kappa light polypeptide; OAS1: 2',5'-oligoadenylate synthetase 1; OASL: 2',5'-oligoadenylate synthetase-like; PBMC: peripheral blood mononuclear cell; PCR: polymerase chain reaction; RT: reverse transcription; SLE: systemic lupus erythematosus; TLR: Toll-like receptor; TNF: tumor necrosis factor.

## Competing interests

The authors declare that they have no competing interests.

## Authors' contributions

H-ML and TM contributed equally to this work. H-ML performed data analysis, interpretation of the microarray studies, sample preparation, stimulating and co-stimulating experiments, RNA purification, quantitative RT-PCR assays, and drafted of manuscript. TM performed data analysis, interpretation of the microarray studies, and patient recruitment. HS assisted with data analysis. CA performed labeling and scanning of the microarrays. YA assisted with data analysis. NY-H assisted with data analysis. KM assisted in microarray data acquirement. NN designed the study, enrolled patients, and assisted with data analysis and interpretation. All authors read and approved the final manuscript.
